# Intestinal Sensing by Gut Microbiota: Targeting Gut Peptides

**DOI:** 10.3389/fendo.2019.00082

**Published:** 2019-02-19

**Authors:** Mihai Covasa, Richard W. Stephens, Roxana Toderean, Claudiu Cobuz

**Affiliations:** ^1^Department of Health and Human Development, University of Suceava, Suceava, Romania; ^2^Department of Basic Medical Sciences, College of Osteopathic Medicine, Western University of Health Sciences, Pomona, CA, United States

**Keywords:** microbiota, obesity, gut peptides, GLP-1, PYY, functional metagenmics

## Abstract

There are more than 2 billion overweight and obese individuals worldwide, surpassing for the first time, the number of people affected by undernutrition. Obesity and its comorbidities inflict a heavy burden on the global economies and have become a serious threat to individuals' wellbeing with no immediate cure available. The causes of obesity are manifold, involving several factors including physiological, metabolic, neural, psychosocial, economic, genetics and the environment, among others. Recent advances in genome sequencing and metagenomic profiling have added another dimension to this complexity by implicating the gut microbiota as an important player in energy regulation and the development of obesity. As such, accumulating evidence demonstrate the impact of the gut microbiota on body weight, adiposity, glucose, lipid metabolism, and metabolic syndrome. This also includes the role of microbiota as a modulatory signal either directly or through its bioactive metabolites on intestinal lumen by releasing chemosensing factors known to have a major role in controlling food intake and regulating body weight. The importance of gut signaling by microbiota signaling is further highlighted by the presence of taste and nutrient receptors on the intestinal epithelium activated by the microbial degradation products as well as their role in release of peptides hormones controlling appetite and energy homeostasis. This review present evidence on how gut microbiota interacts with intestinal chemosensing and modulates the release and activity of gut peptides, particularly GLP-1 and PYY.

## Introduction

The human body has been coined a superorganism for it is the host of a complex consortia of commensal microbes that contain 10-fold more cells and 150 times more genes (1–3). These trillions of microorganisms with thousands of bacterial phylotypes reside mainly in the gastrointestinal tract and are collectively termed the gut microbiota (4, 5). Although its existence and importance has long been recognized, recent advancements in identification, quantification and functional properties of the gut microbes highlights its role in protection against enteropathogens (6–8), extraction of nutrients and energy from our diets (9), and contribution to normal immune functions (10, 11). More importantly, increasing evidence suggests that the cross talk between bacteria and the host is critical in maintaining health and an imbalance (i.e., dysbiosis) has been associated with chronic disorders including obesity (12, 13), diabetes (14, 15), inflammatory bowel disease (IBD) (16, 17), non-alcoholic fatty liver disease (18, 19), malnutrition (20, 21), cancer (22), and psychiatric disorders (23, 24).

Although our understanding of how gut microbiota impacts health and disease is still evolving, the development of quantitative and functional metagenomics offers insights into the mechanisms by which bacteria affects the host in both health and disease. For example, the discovery of novel bacterial metabolites and their activities such as β-D-glucuronidases, neurotransmitters (e.g., cathecolamines) or the new roles of short chain fatty acids (SCFA) in energy balance via gut satiation peptides such as CCK, GLP-1, and PYY underscore the importance of gut bacteria in host metabolism (25, 26).

In this review, we will summarize the most recent findings on the role of gut microbiota in obesity-induced microbiota imbalance. The bidirectional communication between the host and microbes influencing gut chemosensory and metabolic signaling pathways, the current challenges and emerging ideas on using bacteria to change behavior and phenotype will also be discussed.

## Microbiota Signaling via Gut Peptides

The link between gut microbiota and production of gut peptides by enteroendocrine cells has been well documented (27). Enteroendocrine cells (EEC) have been widely studied for their critical role in regulating gut motility, secretion, and production of peptide hormones that control food intake as well as insulin release. Intestinal enteroendocrine cells such as L-cells are strategically positioned to detect the presence of nutrients, microbiota and their metabolites. They act via G-protein coupled receptors (GPCRs) and transporters that activate different pathways known to regulate gene expression and/or to promote exocytosis by raising intracellular Ca^2+^ levels. These open-type enteroendocrine cells are present in high density in the ileum and colon, areas where the majority of bacteria reside. Therefore, there is an intimate relationship between bacteria and eneteroendocrine cells. Not surprisingly, microbiota controls enteroendocrine cells differentiation and the number of GLP- and PYY-secreting L-cells (28). As a result, consumption of non-digestible carbohydrates, prebiotics, direct administration of SCFAs or specific bacteria (e.g., *A. muciniphila*) increases L-cell numbers as well as intestinal expression and release of GLP-2 and PYY, demonstrating a role of bacteria in gene regulation and signaling (29, 30). For example, ingestion of fructooligosaccharides that results in subsequent high production of luminal SCFAs increases proliferation of L-cells that express FFRA2 and GLP-1 (31). This effect involves upregulation of Neurogenin3 (NGN3) and NeuroD, two transcription factors required for enteroendocrine cell differentiation (32). The interactions between microbial metabolites and specialized enteroendocrine cells have an organic, physiological, and behavioral correspondent. For example, decrease in abundance of specific bacteria such as *A. muciniphila* that produce bioactive metabolites with an effect on gut hormones is associated with increased gut permeability, obesity and type 2 diabetes whereas restoration of this bacteria levels reverses such effects (33). Further, blockade of GLP-2 receptors abrogates prebiotic-induced improvements in gut barrier functions (29, 30) demonstrating a causal relationship between microbiota and hormone secretion. GLP-2 receptor has been implicated in regulating intestinal epithelium integrity, and bacteria-induced increase in GLP-2 levels can protect against inflammation (29, 34–36). Notwithstanding the absence of precise regulatory mechanisms, it is clear that bacterial metabolites are active participants in the connection between the enteroendocrine cells secretory milieu and overall host metabolic functions.

**Figure 1:**
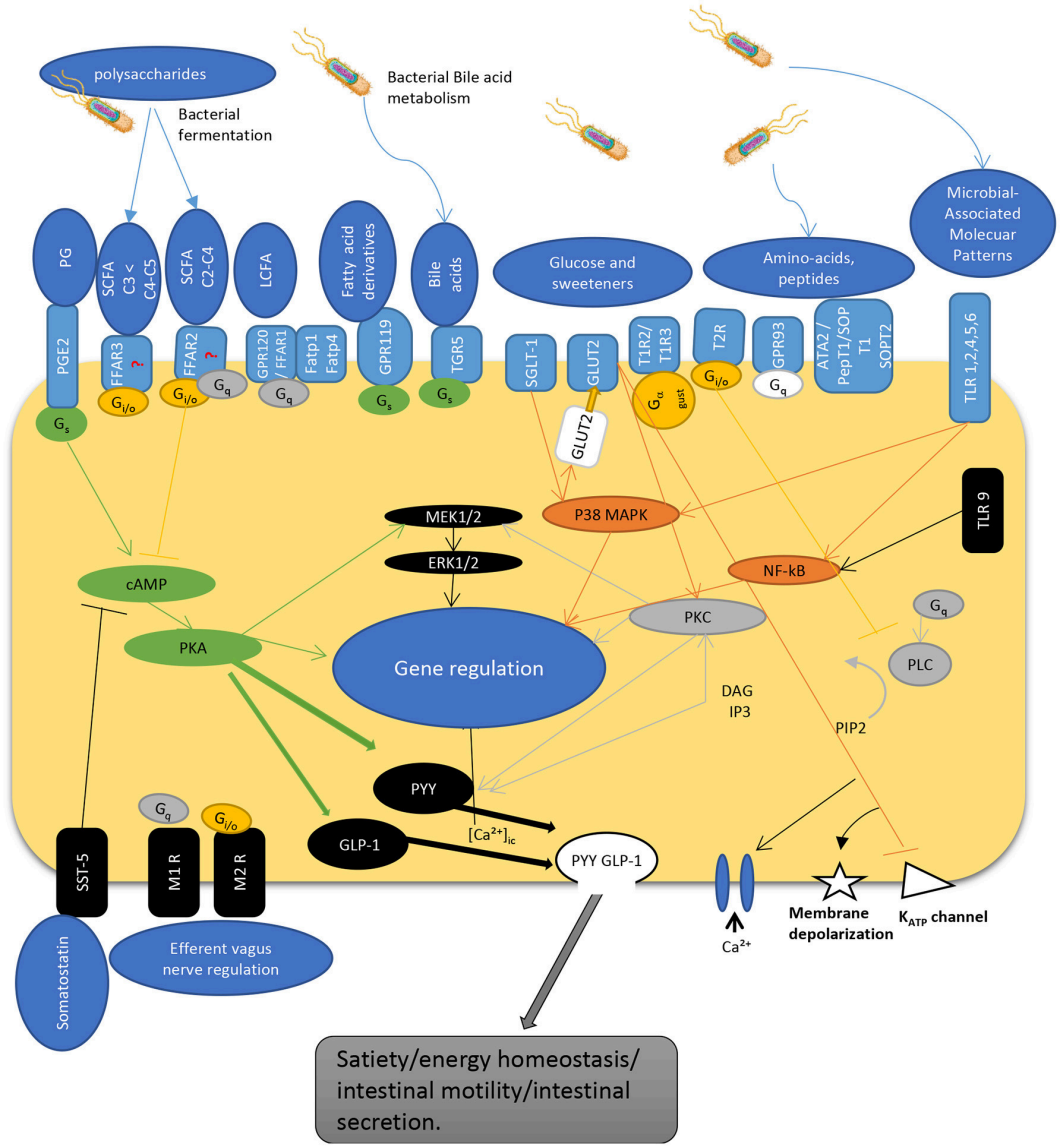
Schematic overview depicting regulatory pathways for PYY and GLP-1 secretion by enteroendocrine L-cell. The L-cells express a wide variety of GPCRs that sense luminal content including dietary and bacterial products. SCFAs are recognized by FFAR2 and FFAR3 expressed at the apical and basolateral membrane [but see (47)]. G_s_ activates adenylyl cyclase, increases cyclic AMP, activation of PKA that regulate gene expression, and activates GLP-1 and PYY. G protein G_i/o_ inhibits G_s_ cAMP pathway while activating PLC pathway. Similarly, G_q_ activates PLC pathway to hydrolysate PIP2 into DAG and IP3. IP3 induces intracellular Ca^2+^ release mediated by voltage-gated Ca2+ channels. DAG activates PKC, an important regulator of cell activity and gene expression. Hormone secretion is also stimulated by the Gs-coupled GPR119 and TGR5. TLRs sense microbial molecules and mediate inflammatory responses through NF-kB pathway. Glucose sensing implicate transporters that act through inhibition of K_ATP_ channels inducing membrane depolarization and calcium entry into the cell. These pathways enable L-cells to communicate with other cells by secreting gut peptides like GLP-1 and PYY, as well as by regulating their expression. PG, prostaglandins; SCFA, short chain fatty acids; LCFA, long chain fatty acids; TLR, toll-like receptor; PGE2-R, prostaglandin E2-receptor, FFAR, free fatty acid receptor; GPCR, G-protein coupled receptor; FATP, fatty acid transport protein; TGR, membrane-type receptor for bile acids; SGLT, sodium glucose transporter; GLUT, glucose transporter; T1R/T2R, taste receptor; ATA, aurintricarboxylic acid; pepT, peptide receptor; SOP, serine-o-posphate receptor; cAMP, cyclic adenosine monophosphate; G_s_, G_i/o_, G_q_, G-protein subunits; MEK, mitogen-activated protein kinase kinase; ERK, extracellular signal-regulated kinase; MAPK, mitogen-activated protein kinase; PKC, protein kinase C; NF-kB, nuclear factor kappa beta; PLC, phospholipase C; GLP-1, glucagon-like peptide; PYY, peptide YY; DAG, diacyl glycerol; IP3, inositol triphosphate; PIP2, phosphatydilinositol (4,5) diphosphate; K_ATP_, adenosine triphosphate sensitive potassium channel; SST, somatostatin receptor; M1R/M2R, muscarinic receptor; [Ca^2+^]_ic_, calcium internal concentration.

The interaction of gut microbiota is not limited to L-cells and their products. Numerous bacteria such as *Lactobacillus, Bifidobacterium, Escherichia, Enterococcus, and Truchuris* among others, interact with other enteroendocrine products such as serotonin and/or produce a large repertoire of their own bioactive molecules including serotonin, dopamine, gamma-aminobutyric acid (GABA), brain derived neurotrophic factor (BDNF), and norepinephrine (25, 26, 37–39). In fact, the enterochromaffin cells (EC) which are the most numerous cell type among the enteroendocrine cells, are the main source of serotonin. They are directly exposed to microbial products and express chemosensory receptors for a variety of microbial metabolites, including short chain fatty acids (40–42). Recent studies in humans and mice demonstrated that gut microbiota promote colonic Tph1 (tryptophan hydrolase 1, the rate limiting enzyme for 5-HT biosynthesis) expression and 5-HT production following stimulation of EC cells by SCFA, such as butyrate and acetate (42). Although it seems that EC cells do not express GPR41 (43) and GPR43 (44, 45) receptors, treatment of human BON cells, a EC model of 5-HT synthesis, with butyrate enhanced *Tph1* transcription in mice via a ZBP-89 zinc finger transcription factor involved in the secretion of antimicrobial peptides (46). Furthermore, it was recently reported that GLP-1 receptor is highly expressed in EC cells and stimulates 5-HT release (26). Along with this, colonic EC cells express increased expression of a host of other receptors sensing microbial metabolites such as FFAR2 and OLFR78 for SCFA, in line with their stimulatory effects on Tph1 expression and 5-HT synthesis; OLFR558, receptor for branched SCFA; GPBARR1/TGR5 for secondary bile acids; GPR35 for small aromatic acids, and GPR132 for lactate and acyl amides (26). Not surprisingly, the expression of these receptor sensing microbial metabolites were much lower in the EC cells of the small intestine where microbial flora is less abundant. As such, dietary supplementation with insoluble fiber like cellulose, significantly increased the density of EC cells as well as fecal content (31). Together, this data demonstrate that colonic EC cells represent a rich reservoir of specialized receptors and are well equipped to directly sense the microbiota-derived biomolecules. This could well explain why disruptions of gut microbiota have been associated with intestinal pathologies including irritable bowel syndrome and other systemic disorders. Figure 1 depicts the different known pathways implicated in PYY/GLP-1 expression and secretion in L-cells in response to luminal molecules.

## Microbial–Derived Short Chain Fatty Acids Serve as Sensing Molecules

It has been well established that SCFAs are the primary candidates in the crosstalk between bacteria and the host. The major SCFAs produced as a result of anaerobic bacterial fermentation of carbohydrates and proteins are acetate, propionate and butyrate. However, other SCFAs, albeit in smaller quantities, such as formate, valerate, caproate, isobutyrate, 2-methyl-butyrate, and isovalerate are also produced from the breakdown of branched-chain amino acids (48). SCFAs, represent an important source of energy for colonic epithelium. These fermentation byproducts also play a regulatory role in energy metabolism of the host providing ~10% of the daily caloric input (49, 50). SCFAs are predominantly present in the colon at high concentrations although, low, micromolar levels can also be found in the circulation (51, 52). SCFAs are also used as substrate by the liver in cholesterol synthesis such in the case of acetate or for liver gluconeogenesis such in the case of propionate. Several bacteria have been identified through metagenomic analyses contributing to SCFAs production. For example, *Akkermansia municiphilla Bacteroides, Clostridium, and Bifidobacterium* are key acetate producing bacteria (53–55) while a small group of bacteria represented by *Faecalibacterium prausnitzii, Eubacterium rectale, Eubacterium hallii*, and *R. bromii* contribute to a large portion of butyrate production (56). Gram-negative bacteria such as *Bacteroides, Veillonella, Dialister*, and *Salmonella* as well as *Coprococcus, Roseburia*, and *Ruminococcus* which are *g*ram-positive bacteria, are mainly responsible for propionate production (57). However, it has been difficult to assess how the interaction between diet and microbiome composition can influence the type and proportion of SCFAs produced in human subjects, although it is rather clear that a diet-induced diverse microbiota results in significant and corresponding changes in SCFA with protective metabolic and health effects. For example, *Akkermansia municiphilla* is less abundant in obese and type-2 diabetic mice and daily administration of *Akkermansia municiphilla* for 1 month reversed high-fat diet-induced obesity and type 2 diabetes (33, 58). Further, diets low in fat and high in fiber content have a “butyrogenesis” effect, thereby increasing butyrate-producing organisms such as *Clostridium symbiosum, Roseburia intestinalis, and Eubacterium rectale* (56). By contrast, absence of the saccharolytic fermentation following consumption of low fiber, high fat feeding, that are indicative of low microbial diversity and butyrate generating bacteria increases inflammatory markers such as CD3^+^ intra-epithelial lymphocytes and CD68^+^ lamina propria macrophages, underlying pathological conditions with inflammatory underpinning (59). SCFAs play a significant role in regulation of tight junction proteins such as claudin-1, Zonula Occludens-1 (ZO-1) and occluding, that are critical in maintaining the integrity of intestinal barrier (60). For example, butyrate restores altered expression of tight junction proteins thus inhibiting macrophage activation and production of pro-inflammatory cytokines (61). Furthermore, SCFAs function as signaling molecules that regulate lipid and glucose homeostasis in an adenosine monophosphate-activated protein kinase dependent manner via peroxisome proliferator-activated receptor-*y* (PPAR-y) (62). Both propionate and acetate have been shown to reduce lipolysis, intrahepatic triglycerides and free fatty acids as well as improving insulin response in pancreatic β cells (57, 63, 64). Finally, microbiota-derived SCFAs have a major role in signaling gut hormone functions. To this end, the discovery 15 years ago that the orphan G-protein-coupled receptors, GPR43 and GPR41 (subsequently renamed FFAR2 and FFRA3, respectively), and later joined by the GPR109A, as well as intracellular signaling molecules inhibiting Lysine/Histone Deacetylase (HDACs), were activated by SCFA, paved the way into examining their role in controlling food intake (65). Activation of these receptors by SCFAs results in a reduction of cAMP production and stimulation of ERK (extracellular signal-regulated kinase) pathway via Gα_i_/_o_ mechanism or by increasing intracellular Ca^2+^ levels and stimulation of MAPK pathway (mitogen-activated protein kinase) via G_q_ family (47).

The findings that FFRA2 and FFRA3 are co-expressed with GLP-1 and PYY releasing enteroendocrine L-cells has raised the possibility that SCFAs may stimulate release of these hormones. Indeed, SCFAs have been shown to increase the number of L-cells in intestinal organoids of human and murin origin (66) and several studies showed that prebiotic and probiotic treatment or direct administration of SCFA increase circulating GLP-1 and PYY (47, 67–72). For example, administration of the probiotic VSL#3 stimulates butyrate production and subsequent GLP-1 secretion (72). Similarly, intra-colonic infusions of propionate increased both circulating plasma GLP-1 and PYY levels but it had no effect on FFRA2 knockout mice (71). This is further supported by data showing that FFRA3 knockout mice exhibit altered PYY expression (47). In as much as these findings implicate SCFAs in production and release of gut peptides known to influence food intake, dietary approaches to increase specific SCFAs aiming at reducing caloric intake and body weight in humans, so far have yielded mixed results. Nevertheless, it is clear that SCFAs are not only critical metabolic substrates for host cellular metabolism but also serve as important regulatory signals in the crosstalk between the bacteria and the host. In addition to responding to bacterial metabolites such as SCFAs, the colonic L-cells are stimulated by indole, a product of bacterial tryptophan metabolism involved in inter-bacterial communication (73–75). Several bacteria from *Escherichia, Bacteroides*, and *Clostridium* genera, among others, have been involved in production of indole that acts on voltage-gated K^+^ channels, enhancing Ca^2+^ entry thus stimulating GLP-1 secretion (73). Finally, gut microbiota may also impact gut hormone secretion through an indirect pathway involving bile acid metabolism. Two important receptors, farnesoid X receptor (FXR) and G protein-coupled bile acid receptor-1 (Gpbar-1, aka TGR5) that regulate bile acid metabolism, glucose and insulin sensitivity are co-expressed in the EECs. Both FXR and TGR5 are involved in bile acid-stimulated GLP-1 secretion (76, 77). This effect was demonstrated in a recent study where intestinal FXR played a critical role in the prevention of diet-induced obesity and diabetes through modulation of gut microbiota (78). As such, treatment with fexaramine, an FXR agonist increased abundance of bile acids-producing organisms. *Acetatifactor* and *Bacterroides*. The resultant increase in bile acids and dehydroxylase activity stimulates TGR5 signaling leading to GLP-1 secretion. Conversely, activation of FXR by gut microbiota reduced bile acid synthesis enzymes and inhibited expression of gluconeogenic genes (79, 80). *Acetatifactor* belongs to the *Clostridum cluster XIV* that is closely related to *Ruminococcus, Clostridum, Anaerostipes*, and *Blautia*. These studies demonstrate how host intestinal signaling constituents can shape gut microbiota to regulate metabolic homeostasis (78).

## Bacteria-gut Peptides Crosstalk: Role of PYY

There is now compelling evidence demonstrating a role of PYY in the control of food intake in both humans and animals (81, 82). Circulating levels of PYY increase after a meal and administration of PYY or stimulation of its secretion reduces food intake and increases sensation of fullness (83). Secreted in proportion to caloric load and meal nutrient content, PYY is synthesized and released mainly from the enteroendocrine L-cells present in the ileum and colon. PYY is secreted during fasting state as well as during the presence of nutrients in the proximal intestine via an indirect pathway through release of CCK or by direct contact of nutrients in the distal intestine. PYY is present in two different isoforms, the full-length PYY(1-36) and the truncated form PYY(3-36), resulting from cleavage by the Dipeptidyl peptidase IV. Degradation of PYY(1-36) into PYY(3-36) is an important step for control of food intake. For example, administration of PYY(3-36) or PYY(1-36) isoforms, both decrease food intake in rats, however in rats lacking DPP-IV, only PYY(3-36) reduces food intake suggesting that the cleaved form is the only one implicated in the control of food intake (84).

PYY exerts control on several GI functions in the interest of maintaining energy balance, nutrient availability and exchanges through the intestinal epithelium. As such, PYY delays gastric emptying (85), reduces gastric and intestinal acid secretion (86), reduces gastrointestinal transit via the ileal brake (87), but enhances it in the colon through a paracrine pathway implicating PYY(1-36) (88, 89) and has an effect on cholesterol intake (90). Generally, PYY increases physiological feeling of fullness by reducing intestinal transit and therefore decreases food intake. PYY also controls nutrient absorption by increasing the contact time of nutrient with the gut epithelium and by reducing exchanges through the intestinal barrier and diminishing secretion of enzymes implicated in digestion and absorption of nutrients. Control of PYY secretion is therefore important for maintaining metabolic balance in humans and other mammals. In addition to its major role in regulating energy intake and appetitive behavior (91) PYY is implicated in other physiological functions including inflammation (92), cell differentiation (93), and proliferation (94). PYY has a low incretin effect as PYY(1-36) inhibits glucose-induced insulin secretion on pancreatic Langerhans islet cells by acting on Y1 receptor. Although PYY(3-36) is not implicated directly in control of insulin secretion, it enhances insulin sensitivity and improves glucose disposal (95). Therefore, augmenting PYY levels may have a positive impact on improving insulin tolerance in obesity related comorbidities.

## Regulation of PYY Secretion and Expression

As mentioned earlier, open type enteroendocrine, such as L-cells, act as sensors of luminal contents (96) through a variety of different receptors, mainly G-protein coupled receptors (GPCR) (97), and transporters. Therefore, they are able to detect a long list of compounds such as: SCFA via free fatty acid receptors FFAR3, FFAR2 (45), and fatty acid transporter FATP4 (98), long chain fatty acids (LCFA) via GPR120 (99), and FFAR1 (100), fatty acid derivates such as oleoylethanolamide via GPR119 (101), primary bile acids through TGR5 (102), peptides and amino acids through GPR93 (103) and Na+ coupled amino acids transporters (104), saccharides via SGLT-1 (105), GLUT2 (106), and taste receptors T1R2/T1R3 (107), bitter compounds through T1Rs (108) and receptors to prostaglandins (109). These receptors activate different pathways like mitogenic p38 MAPK, ERK1/2, phosphatidylinositol 4,5-bisphosphate hydrolysis, or cAMP/PKA activation (110). These pathways are known to regulate gene expression and/or to promote exocytosis by a raise in cytosolic Ca^2+^ concentrations. L-cells also express receptors on their basolateral sides, allowing them to communicate with various organs (111). PYY secretion is also regulated by other gut hormones such as cholecystokinin (CCK) via activation of vagal afferent signaling, which represent the main control of postprandial release of PYY (Figure 1).

## Sensing of Gut Microbiota By Enteroendocrine L-cells

Gut bacteria can influence PYY levels by regulating its gene expression, the number of L-cells, or their secretion. For example, antibiotic treatment has been shown to decrease PYY levels, an effect accompanied by increased food intake and increase in abundance of obesity dominant bacteria (112). Gut microbiota can mediate secretion of PYY either directly through biomolecules and metabolites produced by specific bacteria or indirectly via control of food intake through nutrients that are transformed by bacteria. The direct communication between microbiota and L-cells is mediated through functional toll like receptors (TLRs). TLRs are well studied trans-membrane receptors that participate in the communication between microorganisms and host for different functions such as inflammation or adipocyte regulation (113). Several TLRs, such as TLR 1, 4, 5, 6, and 9 are expressed in mice enteroendocrine cells as well as in STC-1 cells, a model of intestinal endocrine cells that are widely used to study mechanisms of gut hormone secretion such as GLP-1 and PYY. These cells respond to some TLR ligands in classical ways by activating the nuclear transcription factor NF-kB, a critical mediator of inflammatory responses (114, 115). TLR4, 5 and 9 agonists (respectively LPS, flagelin, and CpG) have been shown to increase CCK secretion in STC-1 cells and in *in vivo* models (116). Given that STC-1 cells also secrete GLP-1 and PYY, in addition to CCK, it is reasonable to assume that these bacterial constituents can also induce PYY and GLP-1 secretion. However, whether all types of enteroendocrine cells express these TLRs and whether they regulate peptide expression or secretion is not known. It would be important though to determine whether enteroendocrine L-cells in the *in vivo* models express these receptors and if their activation lead to modulation of PYY gene expression.

## Short Chain Fatty Acids Stimulate PYY

As noted above, the link between microbiota and PYY secretion is well established through production of SCFAs following bacterial fermentation of non-digestible fibers. Activation of SCFA ligands, FFAR3, and FFAR2 induces high levels of circulating PYY (117), however their mediation of SCFAs-induced colonic PYY secretion has been recently questioned (47). FFAR3 and FFAR2 are expressed in colonic L-cells with FFAR3 being mostly activated by butyrate and propionate whereas acetate has higher affinity for FFAR2 (118). These two GPCRs are coupled to different types of G proteins and activate different pathways. For example, FFAR3 is only linked to alpha subunits G_i/o_ which inhibit adenylase cyclase and therefore the cAMP pathway. FFAR2, on the other hand, can also be linked to G_q_ which activates PI(4,5) P_2_ hydrolysis by Phospholipase C into diacylglycerol (DAG) and inositol triphphosphate (IP_3_). DAG is an important regulator of gene expression through activation of protein kinase C (PKC) whereas IP_3_ induces release of calcium from the endoplasmic reticulum, which is the required signal for secretion. Thus, using enteroendocrine L-cells incubated with SCFAs results in secretion of GLP-1 and PYY via different pathways. The effects of SCFAs on PYY secretion were also demonstrated *in vivo* by direct colonic injection of SCFA (119), by using L-cells culture and isolated perfused colon model (47) or by enrichment of diet with various types of fibers, such as inulin, oligofructose (120, 121), galacto-oligosaccharides (122), or arabixylan (123). In a recent study, using an isolated perfused rat colon model that closely mimics the luminal physiological condition of the intact colon, Christiansen et al. showed that colonic-generated SCFAs do not have a strong and potent effect on PYY and GLP-1 release. These authors showed that propionate had no effect on PYY or GLP-1 secretion when administered luminally or vascularly whereas acetate and butyrate increased GLP-1 and PYY secretion to a smaller degree when administered vascularly than luminally and this effect was enhanced by intracellular cAMP. Likewise, fibers can exert different effects on the type and proportion of their byproducts of fermentation (124). Several studies demonstrated that fibers have a marked physiological effect in controlling food intake and weight gain via increase in PYY/GLP-1 secretion (125–127), suggesting that treatment with prebiotics may hold promise in controlling weight gain. SCFAs are mainly produced by specific bacteria and archea, through degradation of polysaccharides, a process that cannot occur in absence of these microorganisms. This was demonstrated in gnotobiotic models (128) where germ-free mice were colonized with a fermentative community composed of *Bacteroides thetaiotaomicron* and *Methanobrevibacter smithii*. Plasma levels of PYY and PYY gene expression were more elevated in mice colonized with both prokaryotes than with single or no colonization and these levels were correlated with increased fecal SCFAs. In germ-free animals known to produce low amounts of SCFA, knocking down the FFAR3 had no effects on PYY plasma levels, indicating the importance of microbiota in activation of FFAR3 pathway. However, recent work by Brooks et al. showed that exposure to fermentable carbohydrates resulted in increase in PYY-cell density and PYY secretion, an effect dependent on FFAR2 by stimulating Pax4, a transcription factor involved in enteroendocrine cell differentiation (129). These effects were mediated by the FFAR2 since FFAR3 agonist had no effect on PYY cell density in colonic organoids (129) although in a recent study using an isolated rat colon preparation, administration of a FFAR3 specific agonist, AR420626, significantly increased PYY secretion (47). Additionally, blockade of FFAR3 did not affect acetate- and butyrate-induced PYY response, suggesting an FFAR3 independent mechanism. These findings led to the suggestion that intracellular mechanisms, rather than FFAR2/3, mediate SCFAs metabolism, which results in activation of Ca^2+^ channels and exocytosis responsible for peptide secretion. Nevertheless, deciphering the exact mechanisms of microbiota-induced L-cell functions and the clinical implications will require further studies.

## Dietary Influences on PYY Production

Long term consumption of dietary fiber caused a marked shift in microbiota composition in obese mice, with a higher count of *Bacteroidetes* and an over representation of specific strains such as *Bifidobacteria, Lactobacilli* (30) and *Roseburia* and decreased *Clostridium leptum* (130). These changes have been associated with higher plasma levels of GLP-1 and PYY (28) as well as with significant upregulation of GLP-1 and PYY mRNA expression (131). A higher count of L-cells in mice following a fiber-enriched diet was also observed (30) which might explain the increase secretory capacity and high GLP-1 and PYY plasma levels without changes in gene expression. Interestingly enough, diets enriched with fibers have been shown to have long lasting effects (132). As such, mice fed with high fiber content diets that results in enhanced colonization with specific beneficial bacteria and changes in microbiota profile exhibit high levels of PYY and are more resistant to the deleterious effects of high fat diets (121). In humans, individuals who consumed a diet rich in fiber for 2 weeks had a significantly higher plasma levels of PYY immediately after breakfast than those who had a normal diet (125, 133). Therefore, the long-term effects of diets rich in fibers on satiation and weight control can be explained by beneficial changes in the composition of microbiota resulting in increased production of SCFAs and subsequent increased secretion of PYY. It should be noted though that not all studies examining *in vivo* effects of dietary fiber and SCFAs supplementation on PYY secretion have yielded positive results (126). Some of these differences have been attributed to variations in the anatomical distribution of PYY-secreting cells between species (134). Bacteria can also influence PYY secretion by controlling the presence and metabolism of amino acids and small peptides as well as by regulating bile acids and glucose levels in the intestinal lumen. Indeed, L-cells express receptors to these molecules, and activation of some of them results in enhanced PYY secretion (27).

It has been documented that germ-free mice utilize significantly less energy from the food consumed and are therefore leaner than conventional mice, despite eating more, an effect likely due to diminished, or absence of, signaling regulating satiety (135). In general, germ free mice are resistant to high fat-induced obesity diet, although some germ free strains may be susceptible to obesity depending on the composition of the diet consumed, its content digestibility and availability for the host (136). Not surprisingly, they have fewer intestinal (but not colonic) endocrine cells compared to conventionalized animals and reduced PYY secretion and gene expression (137). This may be explained by downregulation and reduced activation of GPR40, GPR120, FFAR3, or FFAR2. However, only few mechanisms linking microbiota with secretion of PYY have been reported. This includes the effects of bacteria-generated SCFAs on hormone secretion by activating G_q_ coupled receptor FFAR2 (71, 75, 138) and an enhanced secretory capacity via increased number of enteroendocrine L-cells (129, 139). A direct effect of SCFAs, particularly propionate and butyrate on regulating PYY gene expression has also been reported in rats (140) and in human primary intestinal cultures via HDAC inhibition (141). However, other mechanisms could be involved in the regulation of PYY basal levels and responses to food intake that include L-cells proliferation as deregulation of enteroendocrine cell numbers was reported in some pathologies like lymphocytic colitis (142). Nevertheless, understanding the precise mechanisms in the interaction between bacteria and PYY could lead to developing novel therapeutic strategies to prevent or treat obesity.

## Bacteria-gut Peptides Crosstalk: Role of GLP-1

Glucagon-like peptide (GLP-1) is a signaling peptide secreted from the endocrine L-cells in the distal ileum and colon, alpha cells of the pancreas, and the brain. Release of this peptide is due to the presence of carbohydrates, proteins, and lipids moving along the gastrointestinal (GI) tract. Once secreted, GLP-1 exerts its effects by inhibiting gastric emptying, motility and enzymatic secretions, increasing meal retention, relaxing the proximal stomach, stimulating insulin secretion, and therefore controlling food intake. Due to its importance as an endocrine signaling molecule, controlling its secretion, receptor density, and expression are important in maintaining homeostasis (143). Numerous regulators, such as microbiota, prebiotics and diet, have been shown to play a role in GLP-1 expression and secretion (28).

Similar to PYY, an important stimuli of GLP-1 secretion are microbiota-generated SCFAs (144). For example, luminal applications of propionate at physiological range induced GLP-1 release. However, in a recent study only acetate and butyrate, but not propionate, were able to elicit GLP-1 secretion when administered either in the lumen or vascularly (47). Both FFAR2 and FFAR3 that are expressed by colonic L cells are involved in SCFAs-induced GLP-1 release by increasing intracellular calcium via G_q/11_ and possible G_i_ (145). It appears that FFAR2 are the main contributors to GLP-1 secretion (75) since FFAR2 knockout mice have significantly lower basal levels of GLP-1 or SCFA-induced GLP-1 release than wild-type. However, FFAR3-deficient mice also release low levels of GLP-1 in response to SCFA suggesting that FFAR3 may also contribute to GLP-1 release but to a lesser degree than FFAR2 since FFAR3 agonism did not change the density of GLP-1 cells (129) or GLP-1 secretion (47). By contrast, other studies using colonic crypt cultures showed an increase in GLP-1 secretion following administration of the same selective FFAR2 (Gα_i/_Gα_q_) and FFAR3 (Gα_i_) agonist AR420626 (146), while another agonist AZ1729 acting through similar FFAR2 Gα_i_-signaling had no effect on GLP-1 secretion (147). An increase in GLP-1 secretion was also observed in human NCH-H716 cells treated with FFAR2 selective inverse agonist, BTI-A-404, while propionate, the most potent ligand on rat FFAR, had no effect (70). Therefore, the contribution of FFARs on gut hormone production is still unclear and whether SCFAs can impact release of PYY and GLP-1 in humans and, if so, to what degree, remain to be elucidated.

## Short Chain Fatty Acids Stimulate GLP-1 Release

Germ free animals and mice that were treated with antibiotics and have low levels of SCFAs, exhibit increased fasting plasma GLP-1 (148). This effect was considered an adaptive response of germ free animals by slowing down intestinal transit, due to high energy demands, thus allowing increased nutrient contact and absorption (144). Similarly, lack of microbiota in germ free or antibiotic-treated animals results in GLP-1 resistance and reduced insulin secretion, an effect that was reversed when dysbiotic microbiota was redressed, suggesting that a eubiotic microbial consortium is required for GLP-1 sensitivity (149). This effect was shown to be independent of SCFAs and involved production of microbe-associated molecular patterns (MAMPs) such as bacterial cell wall-derived muramyl dipeptide (MDP) and LPS. As such, GLP-1's effects on insulin secretion is critically impaired in NOD2, CD14, and TLR4 KO mice demonstrating that bacterial recognition is important for GLP-1 sensitivity (149, 150). The nucleotide-binding oligomerization domain (NOD) proteins such as NOD2 are part of the well characterized germ-like encoded pathogen recognition receptors (PRRs), that sense bacterial products and participate in activation and signaling pathways involved in inflammation such as the nuclear transcriptional regulator kappa B (NF-kB). Thus, NOD2 receptors recognize MDPs for gram-positive bacteria like Lactobacilli involved in inflammation. Both MDPs and LPS cause GLP-1 release via production of NO in enteric neurons (151).

As is the case with PYY, increase in GLP-1 secretion was also attributed to an increase in GLP-1 cell density, an effect shown to be independent of FFAR2. The exact mechanisms responsible for microbial byproducts effects on increased GLP-1 cell density are not clear and studies thus far indicate that this is not due to their impact on cell differentiation pathways. Although Cani et al. showed that increases in colonic GLP-1 cell density following fermentable carbohydrates were associated with enhanced expression of transcription factor Neurogenin3 (NGN3), other downstream factors such as Pax4, Pax6, Foxa1, and Foxa2 remained unchanged (32). This indicates that other mechanisms and/or microbiota metabolites may be involved in increased number of GLP-1 cells [see (129)]. GLP-1 secretion is also stimulated by bioactive lipids such as oleoylethanolamide (OEA) that belongs to endocannabinoid-like compounds with a role in control of food intake and regulation of body weight. OEA shares a biosynthetic and degradation pathway with endocannabinoids, and is involved in activating secretion of GLP-1 by enhancing GRP119 receptor located on intestinal L-cells (152). Similarly, acetate, butyrate, oleic acid or lithocholic acid, have all been shown to stimulate GLP-1 secretion when applied to colonic mucosa (145). Finally, the microbiota-derived metabolite indole, known for its role in protecting epithelial cell tight junction and anti-inflammatory properties, enhances GLP-1 release by increasing intracellular Ca^2+^ levels in L-cells (73).

In addition to their well-known role in regulation of energy balance, appetite, gastrointestinal functions, and blood glucose control, the gut peptides such as GLP-1 exert significant anti-inflammatory effects. As mentioned earlier, EEC responsible for incretin hormone secretion such as GLP-1 are also activated by gut microbiota via TLRs generating inflammatory responses. Indeed, proinflammatory cytokines following exposure to bacterial flagelin or lipopolysaccharides (LPS) can impact EEC number and secretion during chronic gut inflammation. Further, GLP-1 receptors are present on immune cells and circulating GLP-1 is augmented during inflammation, supporting a role for GLP-1 in mediating immune and inflammatory responses [see (144), for review]. For example, administration of Exendin-4, a GLP-1 receptor agonist significantly reduced inflammatory cytokines and macrophage infiltration (153, 154). Conversely, inflammatory markers, such as endotoxin, IL-1, and IL-6 provoked GLP-1 secretion in mice, while endotoxin-induced hyperinsulinemia was abrogated by GLP-1 receptor antagonists (155, 156). Finally, increasing circulating GLP via inhibition of DPP-IV activity resulted in suppression of NLRP3 inflammasome, a complex protein involved in activation and maturation of proinflammatory cytokines in several models of inflammatory conditions (157, 158). Not surprisingly secretion of several gut peptides including GLP1, GLP-2, CCK, PYY, ghrelin has been reported to be increased in intestinal inflammation characteristic of inflammatory bowel diseases (26). Together, these results show a reciprocal association between gut peptides, intestinal and systemic inflammation. Considering the major impact of gut microbiota on EEC number and the effect on its metabolites on gut peptide changes, modulation of gut microbiota to influence metabolism and inflammatory pathways through GLP-1 or other peptides may be a promising strategy for treatment of some chronic pathologies including obesity [see (144), for review].

## Dietary Influences on GLP-1 Production

GLP-1 production and secretion is greatly stimulated by fermentable carbohydrates, prebiotics, probiotics and dietary fiber. For example consumption of oligofructose or enhancing microbiota fermentation with prebiotics increases GLP-1 production (159). Prebiotics were also shown to increase colon length and weight resulting in a larger pool of colonic L cells. Furthermore, prebiotic treatment increases abundance of *Faecalibacterium prausnitzii* and *Bifidobacterium* which are associated with increased EEC differentiation, heightened GLP-1 secretion as well as GLP-2-induced trophic effects on maintaining gut barrier integrity (121). Several specific bacterial strains have been shown to regulate GPR120 and GLP-1. In fact stimulation of GLP-1 secretion by microbiota is dependent on intact GLP-1 receptors, since GLP-1 knockout mice or mice treated with GLP-1R antagonist, exendin 9-30, are unresponsive to the effects of prebiotics on insulin sensitivity and food intake (160). Serum GLP-1 levels were also increased by probiotic treatment (161). Mice treated with probiotics had a marked decrease in *Firmicutes* and a significant increase in the genus *Bacteroides*, known for its beneficial effects on adipose tissue lipolysis, increased GLP-1 production and reduced intestinal inflammation (162). Similarly, treatment with probiotics from Lactobacillus such as *L. reuteri* had a significant effect on increasing GLP-1 and GLP-2 in humans without changes in fecal microbiota suggesting a direct effect of *Lactobacilli* on incretin release and inflammation (163). Furthermore, probiotic administration increases expression of proglucagon mRNA and reduced expression of pattern recognition receptors (PRRs) CD-14 and NOD1 that might explain their effect on GLP-1 secretion and improvements in circulating LPS (161, 164). Finally, *A. muciniphila*, the mucin degrading bacteria, has also been associated with increased GLP-1 and GLP-2 cell activity via the endocannabinoid system and 2-oleoylglycerol (2-OG) (33). Thus, it is clear that certain bacteria have the capacity to elicit potent peptidergic responses by interacting with luminal host nutrient-sensing mechanisms. It should be noted however, that animals and humans differ in their response to probiotics, and the microbiota have defense mechanisms to preserve their composition in the face of probiotic treatment. As such, while a significant number of studies show improved results on body weight, fat accumulation and other metabolic parameters in animal models, the results from human trials are still controversial or failed to consistently demonstrate and reproduce such positive effects. Furthermore, most of the results on the impact of probiotics show only associative effects and do not address the causative and mechanistic pathways by which they interact to affect host metabolism. The divergent effects reported thus far between studies as well as between animal and human trials are also due to a host of factors including, but not limited, to the use of small cohorts, lack of long-term studies, large variations in probiotic strain used, single vs. mixed strains, dosage and mode of administration, diet, host metabolic condition, etc. Therefore, capitalizing on some of the positive effects of certain probiotic strains seen in animal models under more restrictive conditions would require an extraordinary effort in teasing apart the specific bacterial and host components that could be targeted in the application of probiotics treatment in human obesity [see (165), for review].

## Gut microbiota: an obesity sensor?

Microbiota has been considered a major environmental factor implicated in the development of obesity. Early pioneering “conventionalization” studies showing that recolonization of germ-free mice with gut microbiota from either lean or obese mice recapitulated the original phenotype highlights the importance of microbiota in obesity. However, these initial ground breaking findings that provided a first glimpse into the potential role of the gut microbiota in metabolic disease have raised more questions than provided answers as to the cause-consequence relationship between bacteria and the host. These early studies investigated the impact of gut microbiota using either genetic, transgenic, or HF-fed models of obesity, neither of which are an accurate reflection of human obesity that encompasses the interaction between genes and the environment. However, subsequent, cumulative studies in both humans and animals have now provided sufficient evidence demonstrating that gut microbiota, and in particular a specific gut microbiota composition, is a causative factor in obesity (12). This was demonstrated by studies showing that changes in microbiota composition following weight loss interventions (dietary induced or after bariatric surgery) are not due to weight loss and that bariatric surgery-induced phenotye is transmissible to the germ free animals. For example, obese patients showed predominance of *Bacteroides* in their microbiota which shifted after gastric bypass in favor of *Prevotella*, a bacterium associated with a healthy diet (166). Furthermore, obesity has been associated with a distinct and differing gut microbial communities compared to lean phenotype and that transfer of “obese” microbiota replicates the obese phenotype of the donor, as well as associated differences in the chemosensory, metabolic, and neural dysregulations (167). While some studies suggest the presence of a specialized “obese” microbiota capable of increased energy storage, most are confounded by the fact that the observed obesity is often resultant from an obesogenic, western diet, known to rapidly alter the gut microbiota (168), thus, making it difficult to ascertain the influence of the host metabolic phenotype vs. the diet on microbial composition during the obese state. Indeed, HF-feeding of humanized gnotobiotic mice results in a rapid shift in microbiome and its metabolic pathways preceding increased adiposity (168) suggesting that direct microbe host crosstalk influences intestinal signaling mechanism. Similarly, genetically obese-resistant mice exhibit decreased Bacteroidetes, and increased Firmicutes during HF feeding, emphasizing that diet and not host phenotype may be the main determinant of microbiota shifts (169). However, the Bacteroidetes/Firmicutes ratio and its usefulness in predicting human obesity has been disputed (170, 171). Nevertheless, several studies showed that “obese” gut microbiota profile is not a mere result of HF-feeding, but instead is unique and conserved to the obese state (172–174). Therefore, this distinct gut microbiota, with phyla, genera, and species-specific differences may be a signature of the obese host phenotype, not only of HF-feeding. This is also consistent with results from the study demonstrating that transplantation of gut microbiota from twins discordant for obesity into GF-mice resulted in increased body mass and adiposity of mice receiving the obese co-twins microbiota compared to the lean co-twin, whether the groups were maintained on a low- or high-saturated fat diet (175). Obese animals as well as humans have levels of bacteria from the *Ruminococcus* genus. *Ruminococcus* is phylogenetically heterogenous, and most species fall under several Clostridium clusters, including *Clostridium cluster IV* and *Clostridium cluster XIVa*. As such, *Clostridium leptum* (cluster IV) has been associated with both obesity and weight loss (176, 177). *Clostridium cluster XIVa* is directly correlated with fat pad mass and BMI (177–179), and contains bacterial species known to break down polysaccharides, promoting monosaccharide absorption, enhanced lipogenesis, and lipid storage (9, 180). One of the most studied bacteria genus with a role in obesity and diabetes has been *Akkermansia muciniphila*, a Gram-negative bacterium of the order Verrucomicrobiales that represents 3–5% of gut microbiota and is the most abundant mucus-degrading bacteria in humans. *A. muciniphila* levels are inversely associated to obesity and diabetes (181, 182) and its administration reduced body weight and metabolic inflammation by reducing plasma lipopolysaccharide (LPS)-binding protein (LBP) and leptin, as well as inactivated LPS/LBP downstream signaling (182, 183). *Akkermansia* is drastically diminished in obese mice and humans but markedly enriched after gastric bypass (33, 184, 185) and in metformin-induced weight loss patients (186). Further, *A. muciniphila* were associated with increased enteroendocrine L-cell activity, and secretion of GLP-1 and GLP-2 (187). Therefore, specific bacteria such *A. muciniphila* can exert a significant impact on mechanisms mediating obesity and diabetes.

Several studies showed a lack of microbial diversity in obese vs. lean individuals and that a high microbiome diversity was correlated with low weight gain and improved metabolic parameters (171). However, it is still not clear whether low microbiome diversity is a cause or a consequence of increased long term weight gain in humans. Taken together, studies examining the host-bacteria relationship in obesity demonstrate the broad and extensive contribution of the “obese” gut microbiota to the modulation of complex molecular signaling machinery responsible for host metabolism, energy storage, intestinal nutrient sensing, and inflammatory pathways. It further demonstrates that humans susceptible to obesity may harbor a disadvantageous gut microbiome that exacerbates adiposity during HF-feeding, that could be used as a potential marker for susceptibility to obesity in humans.

## Microbiota-gut Peptides Crostalk in Obesity

Microbiota presents a high degree of plasticity that can adapt relatively quickly in response to various modifiers such as diet, or bariatric surgery to correct weight gain (188–190). Its composition is subject to quantitative and functional changes by prebiotics, probiotics or even by microbiota transplants. The evidence thus far indicates that obesity is associated with changes in microbiota composition and diversity, as well with dysregulations in gut peptides. However, whether these changes are sufficient for, or direct contributors to, obesity-induced dysmetabolism is not fully elucidated, although significant, corroborative, evidence point us in this direction. Except for invasive, surgical interventions, efforts for sustainable, and long term approaches to treat obesity have largely failed. The gut peptides, ordinarly involved in controlling meal size, have long been of interest as a promising treatment tool to increase satiety, and by doing so, curbing excess caloric intake and reduce weight gain. Among the difficulties in using the gut peptides as effective therapeutic intervention to control body weight is their short half-life time and the complex regulation of their expression and secretion. However, the discovery that gut microbiota impact expression and secretion of peptides such as PYY and GLP-1, has raised the intriguing posibility of “customizing” or engineering a specific bacterial consortium that could sustain the gut peptides' long term inhibitory effects on food intake by controlling their expression and secretion. In fact, several studies have attempted to modify bacterial composition via administration of prebiotics, or probiotics in order to increase specific bacteria or by using fecal transplants, some with promising results (191–193). Although no direct links between specific bacteria and gut peptides have been documented, changes in gut microbiota have been associated with expression and release of these peptides. For example, consumption of a high fat diet increases fasting levels of PYY in young men during 1 week, which corresponds with the timeframe for microbial composition changes. Similarly, elderly anorexia is associated with higher plasma levels of PYY (194), and microbiota changes occur with aging (195). Therefore, manipulation of gut microbiota composition to engineer a specific bacterial consortium or increase abundance of some dominant and beneficial bacteria wih a long lasting effect on the activity of PYY and GLP-1 to inhibit food intake in the interest of maintaining a desirable body weight may prove a viable obesity therapy.

## Microbiota Interact With Other Peptides

In addition to its role in secretion of PYY, GLP-1, and GLP-2, gut microbiota interact with other peptides involved in energy metabolism such as leptin. For example, prebiotic and probiotic treatment reduced sensitivity to leptin-induced inhibition of food intake, body weight, and lipogenesis in obese mice fed a high fat diet (161). The effects on reduced leptin sensitivity by prebiotics were attributed to diminished peripheral and central inflammation, enhanced GLP-1 secretion, and/or to the direct effects of SCFAs on leptin secretion (196, 197). For example, increased *in vivo* levels of propionate and acetate enhanced circulating leptin in mice. These effects appear to be mediated by FFAR2 via G_i/o_ and not G_q_-protein-mediated mechanism, since leptin secretion by SCFAs is blocked by pertussis-toxin, which inactivates G_i/o_ signal transduction (197–199). Furthermore, FFAR3 is widely distributed in both stomach and intestine and co-localizes with a number of gut peptides including gastrin, ghrelin, cholecystokinin, glucose-dependent insulinotropic polypeptide (GIP), secretin, peptide tyrosine tyrosine, and neurotensin (146, 200). This is consistent with the role of SCFAs in triggering release of some of these peptides, although the mechanisms as well as the interaction of bacteria with them remain largely unknown. For example, germ free animals have low levels of CCK and delayed intestinal transit (201, 202). They also exhibit lower levels of plasma ghrelin under basal conditions (201, 203) which increases after fasting. Furthermore, ghrelin levels decrease after prebiotic supplementation (204, 205) or by butyrate, an effect dependent on FFAR3 (206). These effects are negatively correlated with the abundance of certain gut bacteria, including the commensal *Bifidobacterium* and *Lactobacillus strains* (207). Finally, gut microbiota influence gut motility and secretion by interacting directly with intrinsic primary afferent neurons (IPANS) (208, 209).

## Functional Metagenomics: Targeting Gut Peptides

Functional assessement of the microbiome has employed a range of high-throughput omics such as metatranscriptomics, metaproteomics, and metabolomics aimed at providing insights into the complex functional activities and dynamics of groups or individual microbes, their gene expression and the class of microbe-derived biomolecules. Several studies have uncovered selective markers that have been used in intervention trials to understand their functional role at the whole body level. While the use of animal models and techniques employed so far helped in understanding the overall contribution of gut microbiota to host metabolism and establish a causal relationship in obesity, deciphering the mechanistic functions of microbes, their metabolites and their interaction with the host poses numerous challenges and requires a tremendous undertaking.

Current treatment options for obesity are limited, and using gut peptides known to control food intake and regulate body weight as a pharmaceutical tool to prevent overconsumption and weight gain has been of great interest for a long time. The initial discovery that obese individuals harbor a distinct gut microbiota compared to lean people has raised the possibility that the gut microbiota plays a significant role in regulation of body weight and development of obesity in some people. This possibility was further advanced by the findings showing that gut microbes have the ability not only to mimic host hormonal signals, but several peptides resembling native pituitary thyrotropin, insulin, leptin, and MSH, have all been reported to be secreted by microbes [see (210), for review]. There are already several gut peptide mimetics such as GLP-1, DDP IV inhibitors, CCK antagonists, PYY, PP in clinical trials for obesity and diabetes (211). Therefore, detailed functional metagenomics that focus on increasing satiation responses of gut peptides improve their half-life time or modulate signaling to the brain areas controlling appetite and energy regulation, may represent a promising avenue to treat obesity using non invasive interventions.

**Figure 2:**
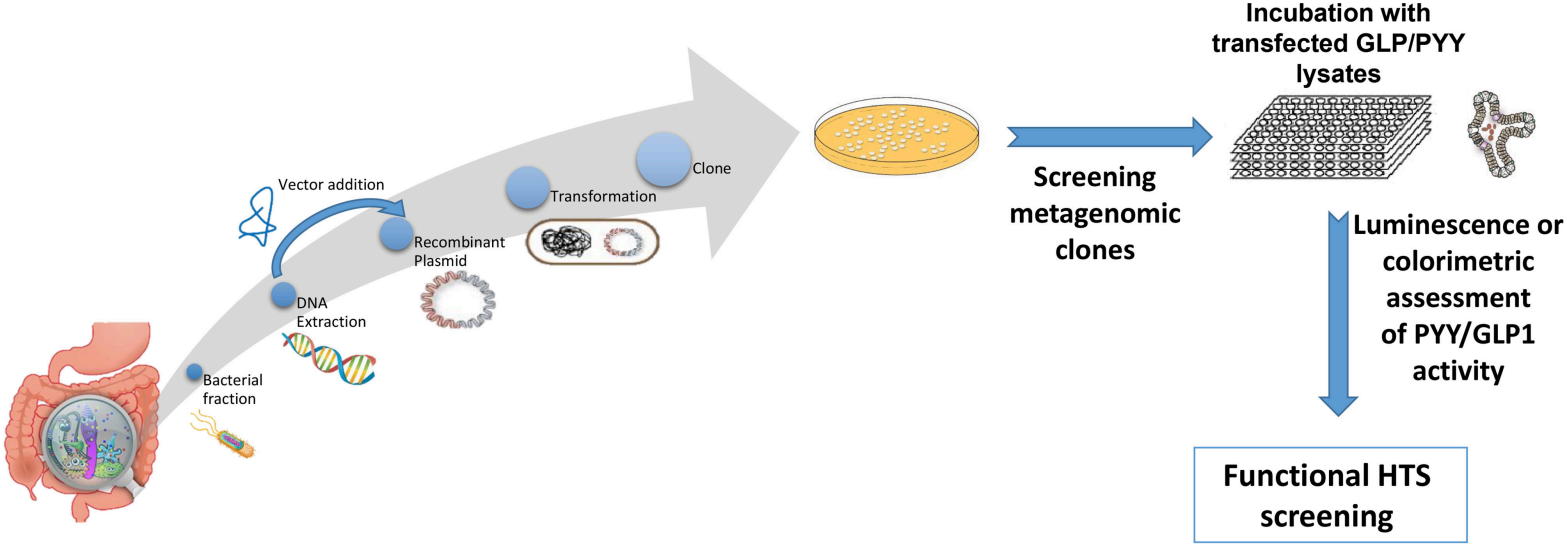
Overview representation of functional metagenomic for screening and identification of bioactive metagenomic clones influencing expression of PYY and GLP-1 from cultured cell lines or enteroid lysates. Briefly, enteroendocrine-like cell lines (e.g., STC-1, NCI-H716) are transfected with a plasmid bearing the promoter region of PYY or GLP1 gene driving a reporter gene (e.g., luciferase; secreted alkaline phosphatase) or a genetically encoded calcium indicator, GCaMP 5G, enabling to follow Ca2+ fluxes, which directly controls peptide exocytosis. Then stably transfected cell clones are selected using selective-resistance techniques and their response characterized. The reporter cells are cultured with each candidate clone from the metagenomic library, to screen for their secretory potential of GLP-1 and PYY. Each metagenomic clone bears DNA of ~40 kb cloned into a fosmid. *E. coli* bearing empty fosmid will serve as control.

**Figure 3:**
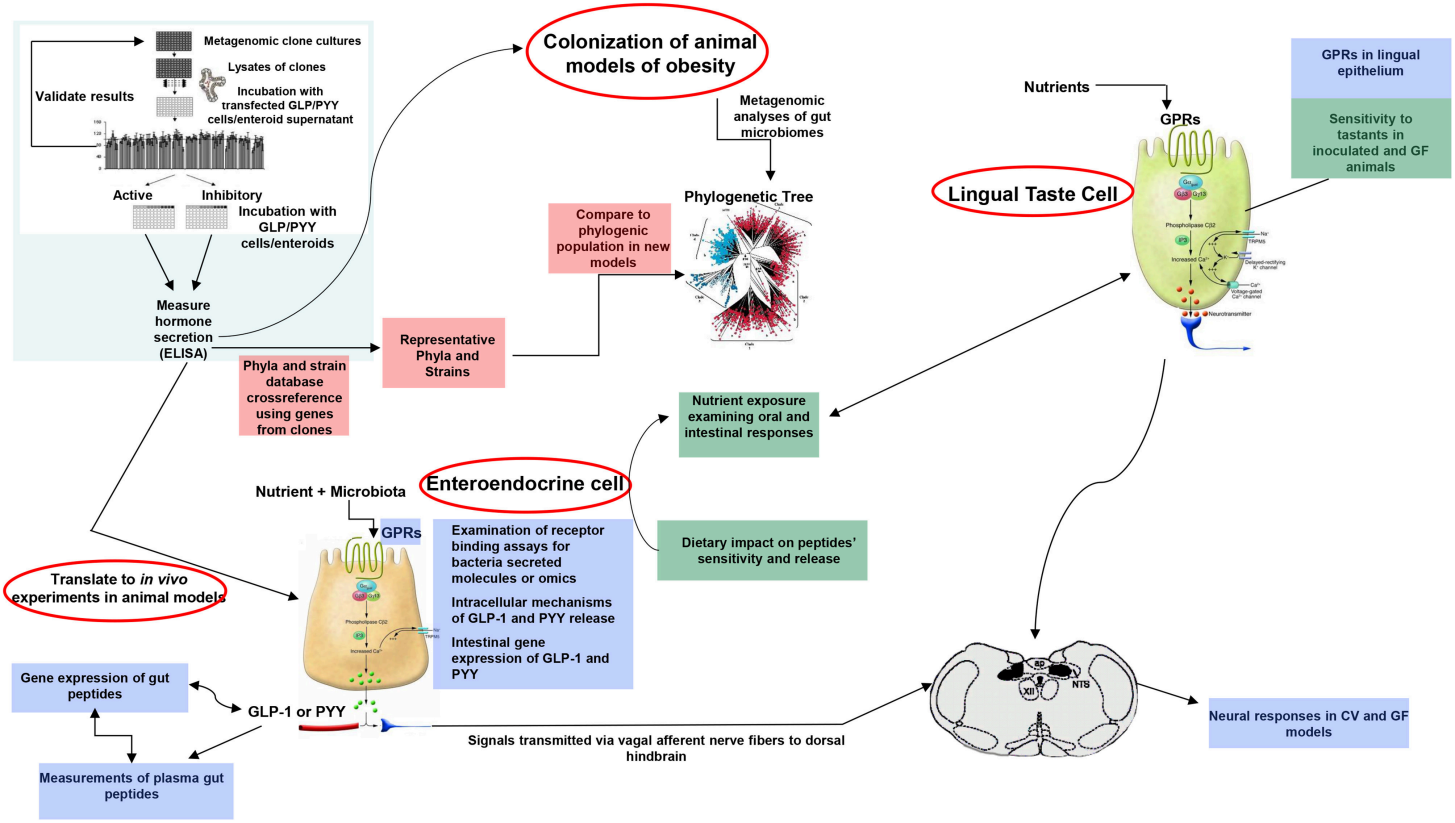
Proposed model of functional metagenomics for studying microbiota-gut peptide interactions. The first step (top left), involves high throughput screening (HTS) of metagenomic libraries to identify candidate clones/bacteria able to stimulate gut peptides (i.e., GLP-1, PYY, CCK) gene expression (using gene reporter technology) and release (following calcium flux) from EEC cultures or enteroids (pink color). The full metagenomic insert of clones of interest is then sequenced to characterize the genes/loci involved using NGS and assign them phylogenetically and functionally. The second step (blue color) represents identification of signaling mechanisms responsible for metagenomic clone-induced-activation of EEC-like cells and gut peptide release. Finally, the effect of identified clones of interest can be tested in obese models for their effects on oral and intestinal contributors to caloric intake, including vagal responses (green color).

A plethora of findings has been revealed regarding changes in microbiota composition profile in obese humans and animals [see (212), for review]. Although some studies have identified specific bacteria implicated in the development of obesity, most studies are descriptive and associative making it difficult to ascertain the causal effect and/or lack the methodolgy capable of addressing the most critical mechanistic questions. Moreover, no specific bacterial strain has been identified thus far as being responsible for obesity development or playing a critical, indispensable role in control of food intake or regulation of body weight. Critical questions still remain unanswered on the degree to which various bacterial metabolites interact with the host chemosensing molecules resulting in a perturbed ecosystem underlying metabolic dysfunctions preceding, or during, obesity. Until now, studies examining functions encoded by the microbial genes that are abundant in the healthy individual but missing in the obese have been limited. Most studies examining the effects of specific bacteria metabolites, such as SCFAs on peptide secretion have used cell line models such as STC-1, GLUTag, HuTu-80, and NCI-H176 derived from adult mouse and human colon adenocarcinoma (68, 213). Altough these cell line models have been extensively used in studying signaling mechanisms involved in hormone secretion, they do not represent an accurate model of native enteroendocrine cells. Therefore, to avoid the limitations inherent with using isolated cell culture models, several studies have employed isolated perfused intestinal segments (i.e., colon), as a model of hormone secretion, which retains the cell polarity and vascular integrity (47). More recently, consideration has been given to the use of organoids (enteroids) that can retain their specificities and regional identity in culture, albeit with some limitations due to their lack of the enteric, lymphatic, and vascular systems (214). However, these models offer great advantage since they are easily maleable and susceptible to manipulation to increase not only the secretory cell capacity but, more importantly, to augment cell proliferation. This latter effect is critical in enhancing hormone release and anorexic response, a model that can be replicated in *in vivo*. In fact, several studies showed that exposure of enteroids with prebiotic-induced SCFAs increased the number of GLP-1 and PYY producing cells (129, 139). A multipronged, integrated approach to examine interactions between gut bacteria and the host is proposed in Figure 3. This process begins with high throughput screening (HTS) of metagenomic libraries and gut bacterial species to identify candidate clones/bacteria capable of stimulating gut peptides gene expression (using gene reporter technology) and release (following calcium flux) from EEC cultures or organoids (see Figure 2). Random transposon mutagenesis can be performed to determine the gene(s)/loci involved in the modulatory effects of the metagenomic clone of interest. Then, transposon insertion sites in the metagenomic DNA inserts can be determined by sequencing, allowing identification of candidate loci/ORF associated with modulation in EEC and assign them phylogenetically and functionally. Molecules involved can be characterized by biochemical approaches as routinely developed for immune-modulatory clones. Secondly, studies can examine the intracellular signaling mechanisms responsible for metagenomic clone-induced activation of EEC and gut peptide release as well as their interactions with nutrient chemosensory signals and the resultant effect on the metabolic phenotype. Finaly, the *in vitro* results can be validated in animals devoid of gut microbiota and obese models in order to test their effects on their potency to reduce caloric intake, promote satiety, and improve/reverse metabolic dysregulation in obesity. Therefore, functional metagenomic analyses can be tailored to address how gut microbiota interact with the host through several pathways involving the enteroendocrine cells. The potential rewards from this inquiry can be major. Since obesity is associated with impaired satiety peptide response, the ability to use gut bacteria to promote endogenous increases in gut peptides which inhibit food intake, could prove efficacious in the fight against obesity and metabolic syndrome.

## Current Challenges in Functional Metagenomics

### Mucosal vs. Luminal Microbiota

Most studies have used fecal samples for metagenomic analyses which raise legitimate questions of whether luminal bacteria may be the best representation of bacterial consortia present at that specific time and point of harvest. The argument is that adherent, mucosal bacteria that comes in intimate contact with the intestinal epithelium and host immune machinery is more stable and maybe more pertinent. However, the answer to this question may not be that straightforward and the relevance of the bacteria sample may depend on the specific study question. For example, fecal bacteria has been characterized by a low microbial diversity and abundance. However, when intestinal microbiota of fecal samples was compared with mucosal microbiota from patients with irritable bowel syndrome there were no significant differences in diversity between the disease and no disease groups (215). Therefore, the challenge still remain in identifying the most relevant disease specific microbiota sampling (enteric vs. luminal), the most appropriate intestinal bacterial regions responsible for the host effects, and, most importantly, in how to capture the dynamic changes at the bacteria/host interface that are associated with particular physiological functions within a given pathology. This may prove an obstinate or even impossible task given the complexity of metabolic disorders such as obesity at both bacteria and host level.

### Causation vs. Correlation

Accumulating evidence thus far demonstrate a clear association between gut microbiota and obesity although, a definite role of gut microbiota in the mechanistic pathways implicated in the development of obesity has yet to emerge. Weight loss interventions via bariatric surgery, dietary, or microbiota modifications have consistently resulted in compositional changes of gut microbiota in both human and animal models. The presence of a microbiota “signature” capable of transferring complex phenotypic traits through fecal transfer represents the most compelling evidence to date of a direct impact of gut microbiota on host physiology. Beyond this major finding, however, our current knowledge and understanding of how bacteria interact with the hosts to bring about consequential metabolic changes is rather limited.

### Translation of Animal Findings to Humans

The complexity of the gut microbial ecosystem with its own structural organization, functions, and metabolic properties that is under the constant influence of the host biological and environmental factors represents a tremendous challenge in the interpretation and translation of animal findings to humans. For example, large variations in the gut microbiota profile due to the animal model used, laboratory conditions, breeding, husbandry, diet composition makes it difficult to faithfully reproduce and generalize the results. Similarly, clinical, observational studies are mainly descriptive and lack the required care either in the experimental protocols or data analyses are of limited use in identifying confounding factors and the source of variability between studies [see (12), for review].

### Experimental Models Used

Gnotobiotic animals have been widely used to interrogate the link between microbiota and obesity. They have been useful in characterizing the microbial communities and examining functional roles of specific strains under controlled host conditions, thus overcoming the challenges inherently present with the human environment. However, useful, germ free animals have a compromised intestinal morphology and physiology, have an undeveloped immune system and are resistant to obesity (216). These functional abnormalities need to be considered when extrapolating the findings to humans or other animal models. In order to avoid some of the confounding factors seen with germ free animals, other alternative models have been used to address specific questions of host bacteria interactions, under various controlled conditions. These include organisms with simplified physiology and circumscribed microbiota such as Drosophila, zebrafish, *C. elegans*, large animal models, non-human primates, and more recently several *ex vivo* organoid models (217–219). It is hoped that the results from these multiple approaches will aid in addressing some relevant clinical questions.

### Methodological Approaches

Another source of variability between studies represents the type of the molecular biology techniques employed (i.e., FISH, DGGE, qPCR, 16S RNA, shotgun sequences), the computational and bioinformatics tools applied, incomplete or lack of data for functional annotations, the type of analyses of metagenomic data sets and, finally, the methods for classification and assembly of metagenomics data employed. In addition, the wide range of study designs and methodological approaches poses a major challenge particularly in studies when a small number of patients are tested that can impact experimental outcomes, data validity and reproducibility. Finally, technical variations in the methodology of fecal collection, fixation and storage methods, DNA extraction protocols, library preparation and sequencing can all affect the accuracy of taxonomic and functional classification that can lead to erroneous inferences when linking specific bacteria with host functions [see (220), for review]. Despite recent advancements in developing novel, sophisticated, high-resolution analyses beyond metagenomics to examine functionality, our current ability to link structure with function across bacteria—host axis in the context of obesity is limited.

## Conclusions and Perspectives

The discovery that gut microbiota may be an important contributor to a host of diseases has generated increased interest and an unprecedented volume of research that at times, can pose a challenge when attempting to delineate the implication of the findings from over interpretation of the data. Not surprisingly, some authors have cautioned about the tendency in the field to explain everything through the microbiota lens (221). Suggestions include using microbiome as diagnostic tool, to monitor patients or to detect diseases (222). While significant strides have been made in implicating the microbiome in some diseases such as Clostridium difficile colitis or Chron's disease or in the development of specific microbial biomarkers, we know very little about the microbiome's role in other pathologies. Notwithstanding the complexity of bacteria-host interaction, it is widely agreed that studies linking microbial functions with a specific host phenotype are in great need and it is critical to determine whether absence or presence of a particular microorganism or consortia of microorganisms alleviate the pathological condition. Thus, it is incumbent that research moves toward identifying functional mechanisms and players involved at both bacteria and host sites. Diversity of the microbiome as well as its richness have been proposed as significant factors associated with optimal health while their absence was aligned with unhealthy outcomes. Since numerous factors shape not only the type and proportion of bacteria in a given environment, but also their functions, it is important to determine microbiome stability in sustaining health, and/or whether manipulating the microbiome is a better strategy to address changes in microbiome-related disease. Likewise, it is critical to learn how specific microbial structures and related functions are shaped by diseases and interventions. In this context, examination of functional redundancies and resilience in various contexts of the perturbed microbiome in pathological conditions is equally important. How can we capture bacteria resilience, transfer it or even develop it to maintain microbiome stability and subsequent health? The multifactorial nature of the disease etiology and the numerous confounding factors makes it difficult to arrive at clear conclusions upon which to design clinical interventions.

Metagenomic analyses have already uncovered numerous microbe producing metabolites and genes; however the challenge still remains in identifying molecules that elicit a strong biological effect and their impact on host mechanisms. Thus, it becomes important that gene isolation and identification is based primarily on functions. There is no doubt that significant progress has been made in our understanding of the importance of gut microbiota in health and disease. However, there is much more that we don't know, and this also includes the role of the remaining and still uncultured microbiota and its associated functions. Establishing disease-related microbiota signatures, understanding the intricate mechanisms through which bacteria metabolites interact with the host signaling machinery with the goal of restoring what constitutes a eubiotic environment to maintain optimal health will bring us closer to microbiota-derived therapeutic interventions.

## Author Contributions

MC, RS, RT, and CC participated in drafting, editing, and writing the manuscript. RS and MC designed the figures. CC, RS, RT, and MC approved the final version of the manuscript.

### Conflict of Interest Statement

The authors declare that the research was conducted in the absence of any commercial or financial relationships that could be construed as a potential conflict of interest.
